# Characterization of irreversible electroporation on the stomach: A feasibility study in rats

**DOI:** 10.1038/s41598-019-45659-1

**Published:** 2019-06-24

**Authors:** Jae Min Lee, Hyuk Soon Choi, Eun Sun Kim, Bora Keum, Yeon Seok Seo, Yoon Tae Jeen, Hong Sik Lee, Hoon Jai Chun, Soon Ho Um, Chang Duck Kim, Hong Bae Kim

**Affiliations:** 1 Division of Gastroenterology and Hepatology, Department of Internal Medicine, Korea University College of Medicine, Seoul, 02841 Republic of Korea; 2 Department of Biosystems & Biomaterials Science and Engineering, Seoul National University, Seoul, 08826 Republic of Korea

**Keywords:** Biotechnology, Biotechnology, Biotechnology, Biotechnology, Gastric cancer

## Abstract

Irreversible electroporation (IRE) is a newly developed non-thermal ablative therapy. During the IRE procedure, the permeability of the cell membrane is irreversibly changed by application of high-energy pulses across the tissue. This induces the breakdown of cell homeostasis, and thereby cell death. Here, we present an *in vivo* study to demonstrate IRE ablation of gastric tissue and characterize the changes that occur with time therein. No significant complications were observed in the test rats during the experiment. The electroporated tissues exhibited apoptosis at 10, 24 and 48 h after IRE ablation. The apoptosis peaked at 10 h after IRE and then declined, suggesting that the ablated tissue rapidly recovered owing to intense metabolic activity. In addition, the electroporated tissues exhibited morphological changes such as pyknosis and karyorrhexis, while histological analysis showed that the blood vessels were preserved. Interestingly, electroporation greatly affected the mucosa and muscularis propria, but not the submucosa and serosa. This study suggests that IRE could potentially be used as a minimally invasive treatment for early gastric cancer that does not exhibit lymph node metastasis or dysplasia.

## Introduction

Electric pulses externally applied to cells induce instability in the lipid bilayer of the cell membrane, followed by formation of numerous nanoscale pores through the membrane, thereby increasing the trans-membrane permeability^1,2^. These electrically induced pores remain in the lipid bilayer for milliseconds to seconds and thereafter are closed once the electric pulse transmission is stopped^3^. Drugs, genes, enzymes, and antibodies can be injected into cells through these transient pores and this technique is called reversible electroporation^4–6^. However, if the energy of the pulses across the cell membrane is increased above a certain electrical field threshold, irreversible pores are formed in the membrane—this is then called irreversible electroporation (IRE)^7^. IRE leads to disruption of cell homeostasis and thereby cell death, without affecting non-cellular components such as the extracellular matrix, blood vessels, and nerves^8,9^.

Since Rubinsky *et al*.^10–12^ proposed that IRE could produce substantial tissue ablation and destroy undesirable tissues in the body, using mathematical and *in vivo* models, numerous papers have been published on the clinical applications of IRE. In particular, IRE has quickly pioneered a new type of cancer treatment, owing to it being an emerging, minimally invasive tissue ablation technology. Recent human pilot studies on malignancies have shown successful anticancer outcomes^13,14^. Moreover, the clinical use of IRE for ablation of breast, pancreatic, and brain tumours; blood vessels; and nerves has shown promising results in animal studies^15–23^.

Early gastric cancer is defined as carcinoma limited to the gastric mucosa or submucosa regardless of the lymph node status^24^. Endoscopic resection is the treatment of choice if the early gastric cancer and absence of lymph node metastasis is amenable to resection^25^. However, endoscopic resection has several drawbacks, including the need for a highly skilled endoscopist, lengthy procedure, and occurrence of serious adverse events such as bleeding and perforation (since the submucosa of the stomach is a thick layer of loose connective tissue that contains blood vessels, lymphatic vessels, and nerves^26^. In contrast, IRE is easy to perform, has a short procedure time, and seems to have a low risk of bleeding and perforation^27–30^. One case study showed that IRE was safe and tolerated by a gastric cancer patient with lymph node metastases^29^. Therefore, if effective ablation by IRE is demonstrated in the stomach tissue as well, the application of IRE for ablating stomach tumours would be an excellent complement to conventional treatment.

Recently, there have been some case reports and clinical studies on IRE in humans^29,31–38^. However, to the best of our knowledge, IRE has not yet been validated for its feasibility and potential use in the ablation of gastric tissue. Therefore, the aim of this study was to show feasibility for the clinical use of IRE in humans through validating the effects of IRE on rat stomach tissues and investigating the pathological changes that occur over time after IRE ablation.

## Results

**Figure 1:**
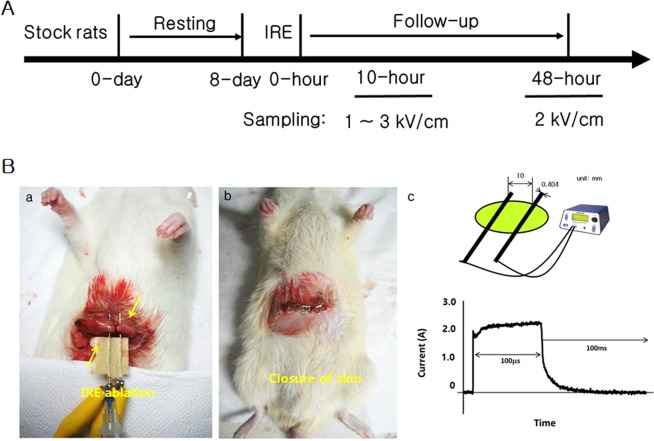
Application of irreversible electroporation within the rat stomach. (**A**) The experimental procedure from housing to observing rats. (**B**) Needle electrodes being inserted into the rat stomach (a), closing of the abdominal incision site using surgical staplers (b) and the connection of electroporator with electrodes and the applied rectangular direct current pulse with a pulse width of 100 μs and a pulse interval of 100 ms when applied at 2 kV (c).

**Figure 2:**
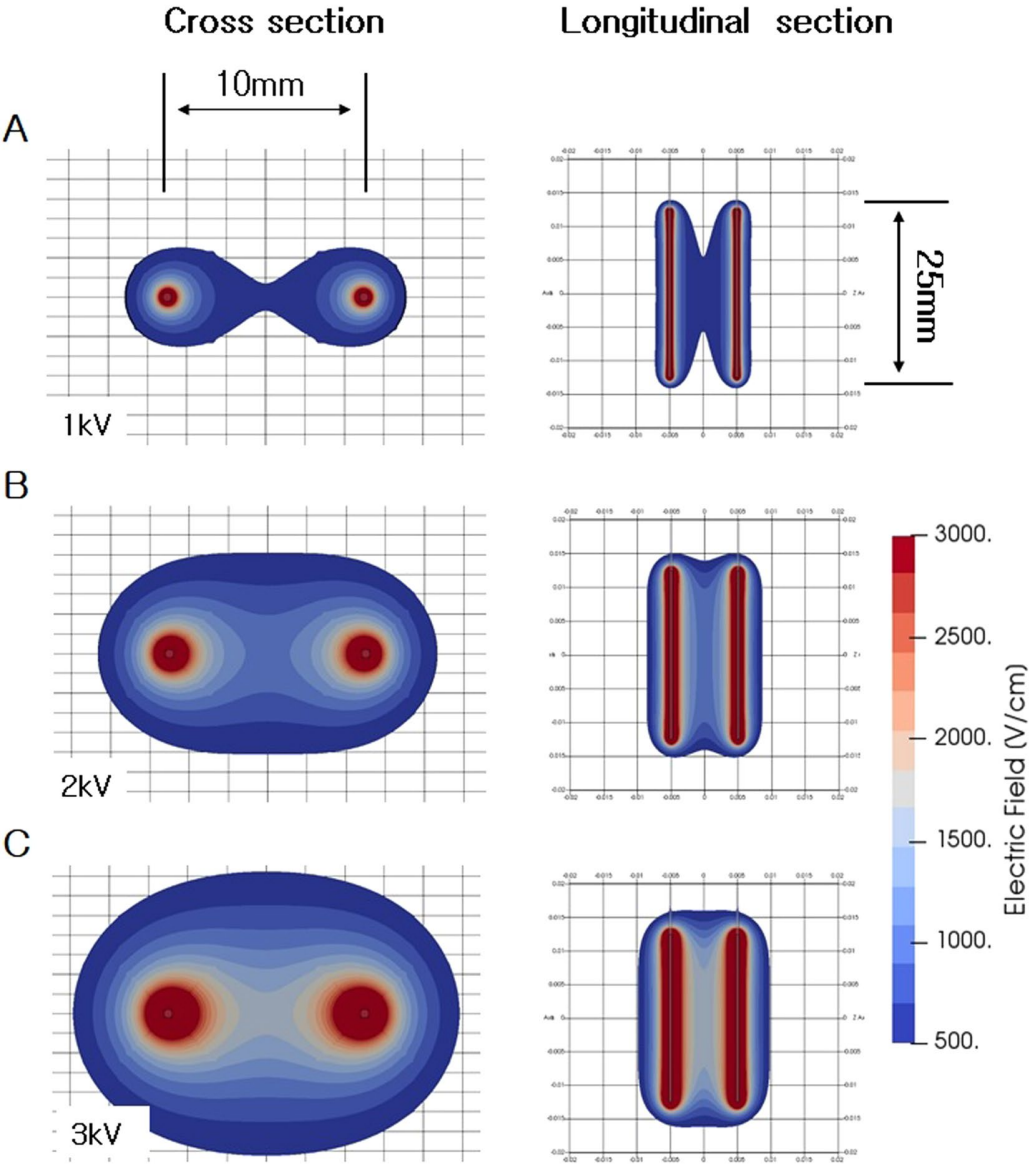
Simulated areas for cross-section and longitudinal section of IRE. (**A**) 1 kV. (**B**) 2 kV. (**C**) 3 kV applied between electrodes (distance 10 mm; exposure length 25 mm). The simulation was performed using open source software called OpenFOAM.

**Figure 3:**
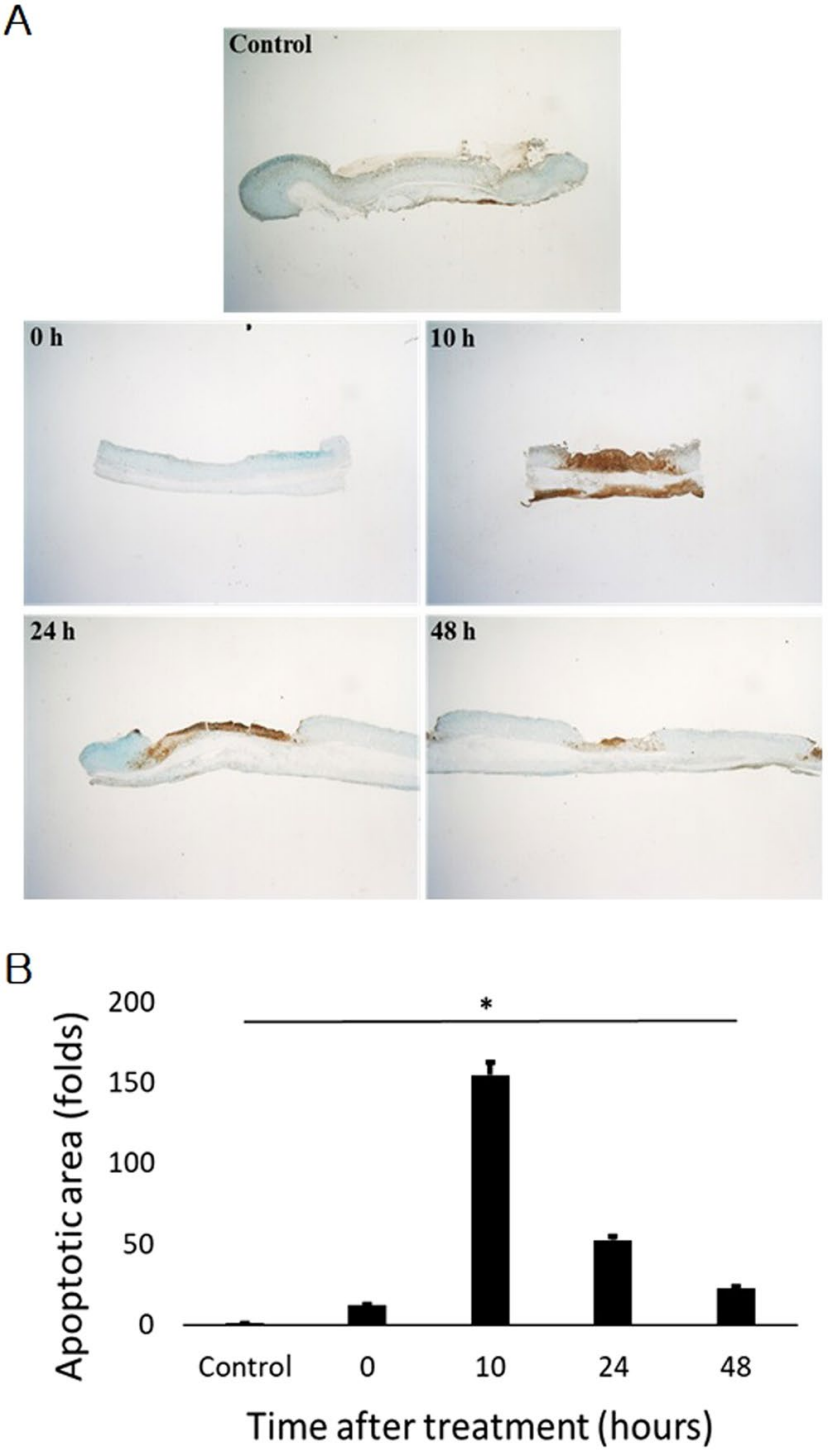
Apoptotic changes with time after IRE (TUNEL assay). (**A**) Apoptosis assay with time. (**B**) The apoptotic area was based on TUNEL assay. Values are the means ± SE (n = 3), *P < 0.05 vs. control.

**Figure 4:**
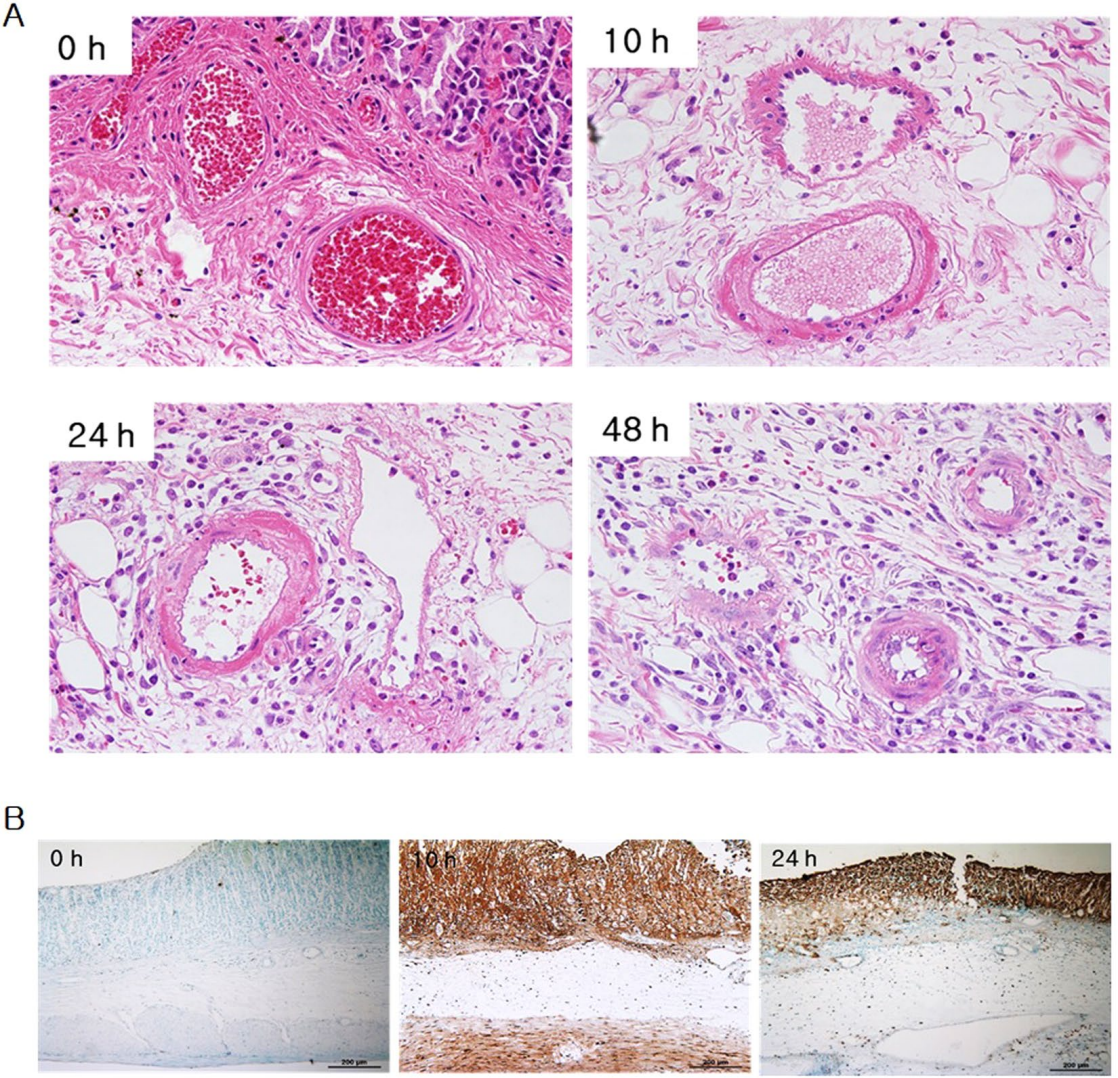
Histological change over time in the stomach after IRE (haematoxylin & eosin staining and TUNEL assay). (**A**) Immediately after IRE, there were no remarkable changes except for subtle mucosal depression and haemorrhage. At 10 h after IRE application, tissue damage was noted in the electroporated area. At 24 and 48 h after IRE application, tissue damage in the electroporated area and neutrophil infiltration throughout the treated area were observed. (**B**) Vigorous apoptosis was observed at 10 h after IRE which correlated with H&E staining results.

For applying irreversible electroporation in the rat stomach, the experiment was designed and set up as in Fig. 1. In order to accurately apply the electric pulses, the distribution of electric field around the electrodes with the external applied voltages of duty cycle 0.1% (pulse width 100 μs; pulse interval 100 ms) was initially calculated. The electric field distribution was dependent upon the external voltages. A strong field distribution was apparent at the centre of the electrode. The electric field was uniform between the two electrodes due to the length of electrodes (25 mm), but vaired at the edges of the electrodes (Fig. 2). We placed the stomach tissue in the region with uniform electric field (Fig. 1B(a)). In study 1, application of 2-kV/cm-50-pulse electroporation resulted in apoptosis, as shown in Fig. 3. As expected, the apoptotic ablation varied with time after IRE. Over time, the colour of the mucosa turned intensely brown and then progressively bleached, while the muscle layer was darkly coloured at 10 h after IRE and very light in colour at 24 and 48 h after IRE. Figure 3B shows the relationship between the apoptotic area and time lapse after IRE. The data were normalized due to subtle difference in distance between electrodes. The maximum apoptotic changes appeared at 10 h after IRE, being about 150-fold compared to that in the control (*P* < 0.05). These changes were significantly attenuated at 48 h through 24 h in comparison with those in the 10-h IRE samples (Fig. 3B). In order to further understand the histological changes occurring over time, H&E-stained samples of the stomach tissue taken immediately and 10, 24 and 48 h after IRE were analysed. On gross examination, no remarkable changes within the treated area of the rats exsanguinated immediately after IRE were observed. However, in the histological analysis of samples taken 10, 24 and 48 h after IRE, the IRE-treated areas presented with dark reddish mucosal defects with round depressions identical to the shape of the electrodes used to apply the pulse. Furthermore, a well-demarcated margin between the ablated zone and the surrounding non-ablated zone was noted macroscopically. The H&E-stained sample taken immediately after IRE, when compared with the 10-h sample, showed acute and severe damage to the tissue in the electroporated area (Fig. 4). Similarly, viable cells were not observed in the tissue samples examined 24 and 48 h after IRE. Instead, neutrophil infiltration was markedly increased throughout the treated area. However, the extracellular matrix and blood vessels, such as small arteries and arterioles within the ablated area retained their original structure (Fig. 4A).

It was evident that the gland structure was largely destroyed, with some haemorrhage within the electroporated area. The margin between the affected and unaffected regions was sharp and corresponded to the applied electrical field (Fig. 4). Notably, the number of dead cells was higher in the mucosa and muscularis propria than in the submucosa and serosa, likely because of their abundant cellular components.

**Figure 5:**
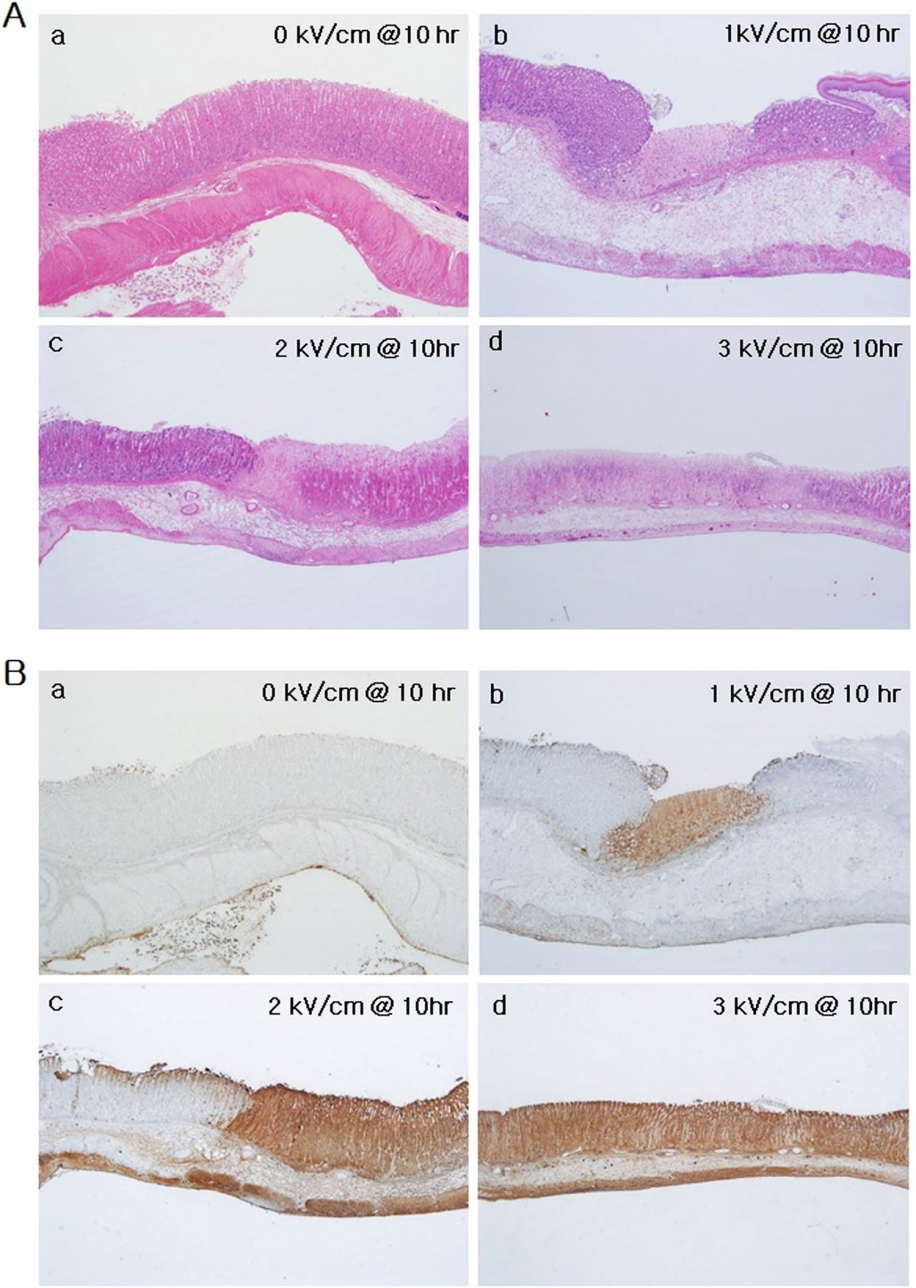
Effects of irreversible electroporation with increasing electric field strength application on the stomach, at 10 h after IRE (H&E and TUNEL assay). (**A**) Non-specific change upon application of 0 kV electrical energy, focal inflammatory change with subtle mucosal depression upon application of 1 kV electrical energy, widespread inflammatory changes and acute tissue damage in the electroporated area upon application of 2 kV of electrical energy, and diffuse inflammatory change and wall thinning in the electroporated area upon application of 3 kV electrical energy. (**B**) Absence of apoptotic area upon application of 0 kV electrical energy is applied, focal apoptosis and cell death observed upon application of 1 kV electrical energy, wide apoptotic area with cell death upon application of 2 kV electrical energy, and diffuse apoptosis upon application of 3 kV electrical energy.

In study 2, which involved IRE application at various amplitudes, all rats recovered fully after the experiment, without significant complications., All the tissue samples were collected at 10 h after IRE treatment, which was the time at which active apoptosis was observed, according to the results of study 1. Differences in the degree of changes in the stomach after IRE were seen among the groups. Figure 5A shows apoptotic changes in the tissues induced by the electrical energy (H&E stained samples). The 0 kV electrical energy did not induce apoptosis. Increasing apoptotic area was observed as the electrical energy was increased from 1 to 3 kV/cm. For IRE at 1 kV/cm, the only remarkable change in the muscularis propria was mild vacuolar formation (Fig. 5A(b)). The submucosa, serosa, and nonablated sites did not show any definite changes. The mucosal defect also went deeper and became more prominent as the electrical energy increased, resulting in cells within the electroporated area becoming uniformly and completely nonviable and showing an extensive degree of pyknosis and karyorrhexis (Fig. 5A(c,d)). Positive TUNEL assay results were observed in the ablated zone, indicating apoptotic cell death (Fig. 5B). As the electric field strength increased, the extent of mucosal damage increased, and wall thinning was observed at 3 kV/cm. The thickness of the mucosa was less at 3 kV/cm than at 1 kV/cm, owing to the shedding of dead cells.

## Discussion

The present study demonstrated that IRE could effectively ablate the stomach tissue through the induction of apoptosis. Moreover, it was revealed that the cellular components were completely ablated by IRE, whereas the extracellular matrix and blood vessels preserved their structural integrity. In this study, cell death due to IRE was uniform and complete within the electroporated area, and it was confined to the area where the electric field was applied. Well-demarcated tissue ablation between the ablated and non-ablated areas at cell-scale resolution is one of the characteristics of IRE, and has been validated in previous studies conducted on other organs^39,40^. In our study, we corroborated that the ability of IRE to precisely ablate tissue as intended holds true for stomach tissue.

The histological changes—cell death, pyknosis, karyorrhexis, destruction of gland structure, and vascular congestion—were observed only in the ablated areas. Additionally, positive TUNEL staining, indicating apoptosis, was restricted to the ablated areas and was absent in the adjacent regions. These results indicate the possibility that IRE is able to selectively destroy an undesirable tissue without collateral damage to the surrounding normal tissue.

In addition, IRE can effectively ablate the target tissue in micro- to milliseconds of treatment time, as compared to the conventional endoscopic resection that requires at least 30 min, and possibly even hours^26^. Once the electrodes are accurately positioned, IRE treatment is completed within a few seconds. Given the shorter procedure time and precise tissue targeting, IRE could be a potential treatment alternative to endoscopic resection for early gastric cancer or dysplasia, with less complications and better outcomes.

**Figure 6:**
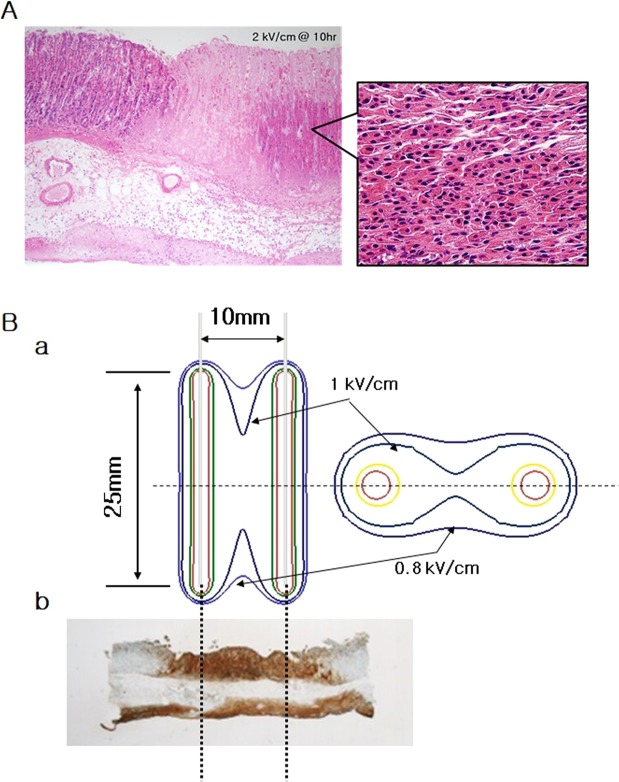
The stomach mucosa (H&E) and the contours of the electric energy density and the shape of ablated tissue according to the theoretical model. (**A**) At 10 h after the application of irreversible electroporation, mucosal tissue was seen to be conserved in the nonelectroporated area. (**B**) The contours predict the ablation area made by the applied electrical energy. The arrow indicates the boundary of the ablated tissue and value of electric field strength is normalized to the critical electric energy density for an ablation of 50 pulses (a). During TUNEL assay, IRE of 2 kV/cm with 50 pulses was applied to the tissue (b).

Apoptosis is a common underlying mechanism by which cell death occurs after IRE.^41^. The apoptosis may not be visible by histological and pathological methods as time passes^42^. This may be related to the apoptotic cell clearance mechanism^43^. The clearance is related to the phagocyte-apoptotic cell interactions that are required for highly efficient removal of dead cells. The engulfment of phagocyte is associated with activation of Rac^43^. When Rac is up-regulated, DNA degradation follows. The DNA fragmentation can be assayed by the TUNEL assay, which is one of the most widely used methods for this purpose^44,45^. As seen in Fig. 3, cells within the electroporated area at 10, 24 and 48 h after IRE stained positively with a dark brown colour in the TUNEL assay. In addition, DNA degradation as revealed by the TUNEL assay peaked at 10 h after IRE, and then declined slightly at 24 h and rapidly at 48 h after IRE^46^. This suggests that the ablated tissue may rapidly recover within these times due to vigorous metabolic activity from the blood circulation^42,43,46^. Here, in Fig. 3A, what seems to be different in distance between electrodes was from which the electrodes were split more or less than 10 mm of distance, when inserting electrodes into the body of stomach. In spite of that, the apoptotic area was well normalized each other in calculation. The apoptotic region may be correlated with the density of the applied electrical energy. From this correlation, we were able to deduce the minimum strength of electrical energy required to induce apoptosis. We calculated the density for the 2 kV electrical energy that was simulated in Fig. 2B, based on the aforementioned Eq. 3. Figure 6B illustrates the areas of ablation contour for 2 kV electrical energy. In the absence of cross-sectional sliced images, it is difficult to calculate the exact threshold of critical energy density. However, we can postulate it to be around 0.8 kV/cm^42,46^. Although the submucosa and serosa were within the applied electrical field, they were not affected. Nevertheless, this study was limited as it evaluated the pathological changes only at 10, 24 and 48 h after IRE. Follow-up studies for long-term outcome are needed to accurately assess the changes after IRE application.

Apoptosis causes morphological changes in the cell. The morphological changes include an irreversible condensation of chromatin material in the nucleus of the cell undergoing apoptosis. A highly extensive degree of pyknosis and karyorrhexis was seen throughout the electroporated area (Fig. 4A) in samples at 10, 24 and 48 h after IRE treatment, but not in samples immediately after pulse treatment. IRE preserves tissue constituents, especially major tissue vasculature since it exclusively targets the cell membrane, and hence only the cellular components^47^. As seen in Fig. 3A, histological analysis showed that the blood vessels were preserved, the extracellular matrix was intact, and only the cells were ruptured and non-viable. Such observations were seen largely within the mucosa and muscularis propria, but not in the submucosa and serosa. (Figs 4B and 6A). According to literature^27^, this is because the gastric submucosa and serosa do not have enough cellular components; however, they are abundant in connective tissues such as collagen and elastin. IRE also caused these things within tissues that were subjected to the energy domain as in Fig. 5.

Interestingly, intact tissue structural components and blood vessels seem to provide a platform for rapid tissue regeneration and contribute to minimal scarring of the treated tissue after IRE^48^, as documented in earlier reports on the liver, prostate, and kidney^49–51^. Rapid tissue recovery after IRE ablation has been reported for the gastrointestinal tract^27,52^. In this study, tissue repair was already evident 3 days after IRE, and the intestinal layers and villi were regenerated within 7 days. The study suggested that the ability of IRE to preserve the extracellular matrix, blood vessels, and nerves aids in the rapid recovery of the small intestine after IRE-induced tissue damage^52^. In fact, delayed tissue healing after treatment seems to be problematic in clinical applications, because it impedes secondary treatment^53^. Rapid tissue recovery after ablation is therefore certainly another advantage of IRE.

This is the first report to validate the effectiveness of IRE on the stomach, however, there are a few limitations to this study that must be acknowledged. This study involved transmural delivery of the electric pulse so as to identify the effect of IRE on the complete gastric tissue. However, this does not reflect real clinical settings, wherein the IRE would be applied through the mucosal or intramucosal route in the gastrointestinal tract wherein the electrodes would be inserted through a channel of an endoscope to deliver the electric pulses. Moreover, this study was performed in normal rat gastric tissue. Since electrical properties, such as impedance, conductivity, and permittivity, differ between cancerous and healthy tissues, the effect of IRE ablation on a normal tissue would be different from the effect on gastric cancerous tissue or dysplastic tissue. In addition, the thermal effect of the pulse treatment was not evaluated. The long-term effect on recovery and regeneration of gastric tissue after IRE also needs to be studied.

## Conclusion

No complications were observed in any animals during the experiments.The apoptotic changes in the gastric tissue as observed through histological techniques and TUNEL assay, seemed to appear at 10 h after IRE and were then attenuated at 24 and 48 h after IRE. The corresponding tissue in an ablated area preserved the extracellular matrix and blood vessels as seen by H&E staining, although neutrophil infiltration was markedly increased throughout the treated area. With application of enhanced electric field strength, the apoptotic area was significantly increased. The extensive degree of pyknosis and karyorrhexis within the IRE-mediated ablated tissue was corroborated at the level of cells. The results of this study could be used to develop strategies for treating gastric cancer or dysplasia through endoscopy combined with IRE.

## Materials and Methods

### Animal preparation

Twenty-seven, six-week-old, female Sprague-Dawley rats (300–400 g) were purchased from Orient. Co. Ltd. (Korea). The animals were acclimatized to the environment by housing them at 22 ± 3 °C in light-controlled conditions (12-h light and dark cycle) for one week (Fig. 1A). The animals were then fasted, but were provided water, for 24 h before the initiation of the experiments. All experimental procedures and animal care were performed in accordance with the protocols approved by the Institutional Animal Care & Use Committee, Korea University (IACUC no. KOREA-2016–0062)

### IRE protocol and experimental set-up

To perform *in vivo* IRE ablation in the rat stomach, the rats are anaesthetized with isofluorane inhalation after medication with muscle relaxants. After skin preparation by shaving and decontamination with an iodine surgical scrub and 70% ethanol, the abdominal wall was dissected to visualize the stomach. Thereafter, two needle electrodes (syringe needle 26 gauge (diameter: 0.404 mm, length: 25 mm) made with stainless steel) with 10-mm separation were inserted into the stomach (Fig. 1B(a)), which has lower conductivity. The two needles were then connected to an electroporator (ECM 830 Electroporator; BTX Genetronics, San Diego, CA), which provides a square wave pulse (maximum voltage, 3,000 V; pulse duration of 10 μsec to 999 ms) via a cable (provided by BTX Genetronics) (Fig. 1B(c)). Here, the applied rectangular direct current pulses had a pulse width of 100 μs and a pulse interval of 100 ms, gained from using an oscilloscope (Protek 3120 with PCP-5000 current probe, Korea) (Fig. 1B(c)). When we apply, the 5-pulse was delivered each at one time and 10 groups in manual to totally be 50 pulses in order to avoid the pulse-resulted thermal effect. However, if an arc was formed between the two electrodes during IRE, we discarded the sample and used a fresh one to repeat the procedure. Th arc occurred one time at applying 3 kV/cm. Following the electroporation, the abdominal incision was closed using surgical staplers (Fig. 1B(b)). The mice were fasted until sacrifice, for collection of tissue for further study. Two different electrical pulses were applied according to the methods of “study 1” and “study 2” (Table 1).

**Table 1:** Experimental design is divided into study 1 and 2.

	E.field (kV/cm)	Pulse number	Groups					Pulse width/interval
Study 1	2	50	Con	0 h	10 h	24 h	48 h	100 μs/100 ms
			n = 3					
Study 2	1 ~ 3	50	Con	1 kV/cm	2 kV/cm	3 kV/cm		
			n = 3					

### Study 1. Comparison of changes over time in the stomach after IRE

In order to investigate the apoptotic changes taking place over time post irreversible electroporation, the constant 2-kV/cm-50-pulse with pulse width of 100 µs was applied to fifteen rats. These were divided into five groups: non-ablated control group (n = 3), 0-h group (n = 3), 10-h group (n = 3), 24-h group (n = 3), and 48-h group (n = 3). Each of them was sacrificed at their set times after irreversible electroporation.

### Study 2. Comparison according to application of various amplitudes for IRE generation in the stomach

Twelve rats were divided into four groups: non-ablated control group (n = 3), 1 kV/cm group (n = 3), 2 kV/cm group (n = 3), and 3 kV/cm group (n = 3). Fifty direct current pulses of 100 µs width were applied with electric field strengths of 1, 2 and 3 kV/cm to investigate the IRE effectiveness of the applied electric field strengths. All rats were sacrificed at 10 h after IRE. These electrical parameters are known to be sufficient to induce IRE in gastric tissue^54^.

### Tissue collection and histological analysis

After IRE treatment, the stomachs were excised and fixed in 10% neutral buffered formalin for at least 24 h. We trimmed the tissues of the IRE-ablated and non-ablated areas. The non-ablated tissues were used as the controls. The tissues were embedded in paraffin and sectioned into 1-mm-thick slices, and then stained with haematoxylin and eosin (H&E) for histomorphological analysis. To verify apoptotic cell death caused by IRE ablation, terminal deoxynucleotidyl transferase dUTP nick end labelling (TUNEL) assays were conducted using an *In Situ* Cell Death Detection, POD kit (Roche, Germany), This method detects DNA fragmentation resulting from apoptotic signalling cascades by labelling the terminal end of nucleic acids. The slides for the TUNEL assay were imaged with an image acquisition system (Olympus BX-51 with digital imaging system (Image-Pro plus 4.5)), and the area coloured brown by TUNEL staining was then analysed via MATLAB (R2018b, 9.5.0.944444, The MathWorks, Inc., Natick, Massachusetts, USA) to quantify the degree of apoptosis in the region of interest.

### Analytical modelling of the ablation area

The applied electric field strength distribution is an important parameter to determine the tissue ablation by IRE. The steady state electric field distribution is obtained by solving the Laplace equation,

1 $${\nabla }^{2}\varnothing =0$$

where ∅ is the electric potential, assuming that the electrodes length is larger than the distance between the electrodes and the effect of tissue surface is neglected^42^. The electric field is calculated as the gradient of electric field potential.

2 $${\rm{E}}=\nabla \varnothing $$

The boundary condition of tissue was defined as ∅ = *V*_0_ or 0. The remaining boundaries were considered to be electrically insulating $(\frac{d\varnothing }{dn}=0)$. We calculated the electric field using Epo code^TM^ (The Standard Co. Ltd., Korea) developed by open source application OpenFOAM for the above conditions.

The electric field is related to electric energy density and thus gives us information about the threshold strength of the electric field required to induce a apoptosis.The applied electric energy density is expressed as^42^:

3 $$w={(E)}^{2}n\Delta t/\rho $$

Where $E=|\mathop{\to }\limits_{E}|$, Δ*t* is the time duration of the electric field application, *n* is the number of the pulses. The curve of points where the electric energy density is the critical value *w*_*c*_ and is the ablated area’s boundary.

### Statistical analysis

Data were analysed using Microsoft Excel. Means and standard deviations were calculated. An unpaired two-sided student’s *t* test was applied to all data. Results were considered to be statistically significant when *P* < 0.05.
