# Publisher Correction: Characterization of irreversible electroporation on the stomach: A feasibility study in rats

**DOI:** 10.1038/s41598-019-54656-3

**Published:** 2019-11-27

**Authors:** Jae Min Lee, Hyuk Soon Choi, Eun Sun Kim, Bora Keum, Yeon Seok Seo, Yoon Tae Jeen, Hong Sik Lee, Hoon Jai Chun, Soon Ho Um, Chang Duck Kim, Hong Bae Kim

**Affiliations:** 1 Division of Gastroenterology and Hepatology, Department of Internal Medicine, Korea University College of Medicine, Seoul, 02841 Republic of Korea; 2 Department of Biosystems & Biomaterials Science and Engineering, Seoul National University, Seoul, 08826 Republic of Korea

Correction to: *Scientific Reports* 10.1038/s41598-019-45659-1, published online 24 June 2019

In the original version of this Article, Hong Bae Kim was incorrectly listed as the corresponding author. The correct corresponding author for this Article is Hyuk Soon Choi. Correspondence and request for materials should be addressed to mdkorea@gmail.com. This error has now been corrected in the HTML and PDF versions of the Article.

